# Análise comparativa de políticas de regulamentação de ensaios clínicos

**DOI:** 10.26633/RPSP.2020.3

**Published:** 2020-01-06

**Authors:** Walter Bataglia, Fernanda Salvador Alves, Eduardo De-Carli

**Affiliations:** 1 Universidade Presbiteriana Mackenzie Programa de Pós-Graduação em Administração São PauloSP Brasil Universidade Presbiteriana Mackenzie, Programa de Pós-Graduação em Administração, São Paulo (SP), Brasil.; 2 Universidade Federal do Paraná (UFPR) Departamento de Administração Geral e Aplicada Programa de Mestrado Profissional em Propriedade Intelectual e Transferência de Tecnologia para Inovação CuritibaPR Brasil Universidade Federal do Paraná (UFPR), Departamento de Administração Geral e Aplicada, Programa de Mestrado Profissional em Propriedade Intelectual e Transferência de Tecnologia para Inovação, Curitiba (PR), Brasil.; 3 Universidade Federal do Paraná (UFPR) Programa de Pós-Graduação em Administração CuritibaPR Brasil Universidade Federal do Paraná (UFPR), Programa de Pós-Graduação em Administração, Curitiba (PR), Brasil.

**Keywords:** Ensaio clínico, política de saúde, legislação de medicamentos, inovação, Brasil, Clinical trial, health policy, legislation, drug, innovation, Brazil, Ensayo clínico, política de salud, legislación de medicamentos, innovación, Brasil

## Abstract

O presente trabalho comparou as regulamentações relativas à realização de ensaios clínicos no Brasil, Índia, Canadá e União Europeia, com objetivo de oferecer subsídios para uma avaliação da política regulatória brasileira. Para tanto, foi realizada uma análise documental em quatro etapas: estudo preliminar exploratório; estudo descritivo; categorização das informações; comparação dos conteúdos. Quanto às distinções entre a regulamentação brasileira e as dos demais casos, merecem destaque a existência de várias resoluções brasileiras sobre o tema, enquanto os demais países concentraram as informações em uma única regulamentação nacional; a não exigência de rastreabilidade dos medicamentos e de recolhimento e descarte dos medicamentos não utilizados quando um estudo é suspenso ou cancelado; e um tempo de aprovação para início do ensaio de 180 dias no Brasil (*versus* 30 dias na União e no Canadá, por exemplo). Isso sugere oportunidades para melhorias e atualização da regulamentação brasileira frente ao cenário internacional.

## Abstract (en)

The present article compares clinical trial regulations in Brazil, India, Canada, and the European Union with the aim of providing evidence for an evaluation of the Brazilian regulatory policy. For that, a document analysis approach was used, in four steps: preliminary exploratory analysis; descriptive study; categorization of information; contrasting contents. Noteworthy differences between the Brazilian regulatory framework as compared to the other regions studied include the existence of several laws and regulations in Brazil vs. a single rule in the other countries; the absence of requirements regarding drug traceability and collection and disposal of unused drugs if a study is interrupted or cancelled; and a higher time of 180 days for approval of clinical trials (vs. 30 days in the European Union or Canada, for example). This suggests opportunities for improvement and update of the Brazilian regulations *vis-à-vis* the international scenario.

## RESUMEN

En el presente trabajo se compararon los reglamentos sobre la realización de ensayos clínicos en Brasil, India, Canadá y la Unión Europea, con el objetivo de ofrecer subsidios para una evaluación de la política de reglamentación de Brasil. Para ello, se realizó un análisis documental en cuatro etapas, a saber, estudio preliminar exploratorio, estudio descriptivo, clasificación de la información y comparación de los contenidos. En cuanto a las diferencias entre la reglamentación brasileña y la de los demás casos, cabe destacar que en Brasil existen varias resoluciones sobre el tema, a diferencia de los demás países donde la información se concentró en una sola reglamentación nacional; no se exige que se rastreen los medicamentos ni que se recojan o se eliminen los no utilizados cuando se suspende o se cancela un estudio; y se requiere un tiempo de aprobación de 180 días para iniciar el ensayo (en comparación con 30 días en la Unión Europea y Canadá, por ejemplo). Eso indica que hay oportunidades para mejorar y actualizar la reglamentación brasileña tal como ocurre en el escenario internacional.

Na indústria farmacêutica, a diferenciação entre produtos exige grande infraestrutura de ciência e tecnologia e altos investimentos em atividades de pesquisa e desenvolvimento (P&D) por parte das empresas (1-3). Para manter a competitividade e a lucratividade, é necessário que haja inovação, num processo complexo, longo e caro (4-6).

Tendo em conta que o desenvolvimento de um novo medicamento pode demorar até uma década entre a descoberta e a comercialização (2, 7), a indústria farmacêutica instituiu a terceirização de determinadas etapas do processo. Com isso, partes do processo foram deslocadas para países da América Latina, do Leste Europeu e da Ásia (7) – especialmente nas fases de ensaios pré-clínicos e clínicos, sem os quais um medicamento não poderá ser comercializado. As estimativas indicam que a porcentagem de medicamentos que chegam ao mercado após o início dos ensaios clínicos varia de 6% a 13,8% (1).

Quando um país tem um ambiente econômico, farmacológico e tecnológico que permite o desenvolvimento de pesquisas clínicas com altos padrões de qualidade, sua relevância científica nacional e internacional cresce, seu potencial de desenvolvimento aumenta e a oferta de saúde pública de maior qualidade para a população se concretiza. O aumento no número de ensaios clínicos realizados num país o coloca dentro de redes globais de conhecimento e de produção de medicamentos e produtos de saúde (1).

Diversos requisitos precisam ser atendidos para que um país seja incluído entre os que participam de ensaios clínicos: conformidade com boas práticas clínicas (BPC), volume de voluntários, tempo adequado entre o recebimento do protocolo e a inclusão do primeiro participante no estudo, custos competitivos, dados epidemiológicos, potencial comercial para o produto no país e regulamentação que viabilize os estudos (8). No entanto, mesmo que a América Latina apresente características que incentivam sua escolha para ensaios clínicos (9), apenas 7,5% dos estudos realizados no mundo ocorrem nessa região (10). A participação do Brasil nas fases I e II, que são as de maior conhecimento, é ainda menor (4). Compreender o motivo pelo qual países com potencial para participar de ensaios clínicos – como o Brasil – são preteridos pode ser benéfico para reduzir entraves. Uma forma de avaliar essa temática é comparar a regulamentação que rege os ensaios clínicos em diferentes países.

Frente a isso, este artigo tem como objetivo comparar as regulamentações relativas a ensaios clínicos em diferentes países e regiões: Brasil, Índia, União Europeia e Canadá. A escolha da Índia se deu pelo fato de ser, juntamente com o Brasil, um dos BRICS, além de ser um país em desenvolvimento com pouca experiência no recebimento de ensaios clínicos. A União Europeia e o Canadá foram escolhidos por fazerem parte do grupo de países desenvolvidos e por terem histórias consolidadas no recebimento de ensaios clínicos.

## ANÁLISE DOCUMENTAL

Neste estudo, a discussão do tema proposto baseou-se em análise documental (11). Estudos desse tipo extraem informações dos documentos analisados, organizando-as e interpretando-as segundo os objetivos da investigação proposta (12).

A coleta de dados foi feita em quatro fases, de março a junho de 2017. A primeira fase foi um estudo preliminar exploratório, que possibilitou reunir documentos sobre regulamentação nos diversos países. Para isso, realizaram-se buscas em *websites* (https://google.com.br/, https://scholar.google.com.br/, https://www.emergogroup.com/, https://www.centerwatch.com/, http://www.clinicaltrialsarena.com/) utilizando o nome do país e as palavras-chave *clinical trial*, *legislation* e *health ministry*, entre outras. Nessa fase, informações dos *websites* regulatórios dos países também foram colhidas – no Brasil, foram consultados os *sites* da Sociedade Brasileira de Profissionais em Pesquisa Clínica (http://www.sbppc.org.br/portal/index.php), do Registro Brasileiro de Ensaios Clínicos (ReBEC) (http://www.ensaiosclinicos.gov.br/) e da Agência Nacional de Vigilância Sanitária (http://portal.anvisa.gov.br/); no Canadá, o site do Centro de Ensaios Clínicos (http://clinicaltrialscanada.com/) e dos Institutos de Pesquisa (http://www.cihr-irsc.gc.ca/e/193.html); na União Europeia, sites relacionados a ensaios clínicos em diversos países (https://ec.europa.eu/health/human-use/clinical-trials/regulation_en; http://www.dli.dk/Pages/welcome.aspx; http://www.regioner.dk/services/in-english); e na Índia, site do Conselho de Pesquisa Médica (http://www.icmr.nic.in/) e do registro de ensaios clínicos no país (http://ctri.nic.in/Clinicaltrials/login.php). Também foram consultados https://www.who.int/ictrp/en/; http://www.ifpma.org/; e https://www.fda.gov/Regulatory-Information.

Na segunda fase, um estudo descritivo foi feito a partir da leitura das regulamentações, dos *websites* e dos documentos obtidos, iniciando com a documentação brasileira pela afinidade com a língua vernácula. Na terceira fase, foram criadas as seguintes categorias para análise: responsabilidades de patrocinador, promotor, investigador; documentação; qualidade e inspeção; prazos; alterações, modificações e emendas; suspensão e cancelamento; efeitos adversos; instalações dos centros de ensaio clínico; importação; e relatórios. Finalmente, compararam-se, na fase quatro, os conteúdos identificados em cada categoria, destacando similaridades e distinções entre os países e a região analisados, a fim de desenvolver subsídios para a melhoria da política regulatória brasileira.

## REGULAMENTAÇÃO

No Brasil, existem diversas normas relacionadas à realização de ensaios clínicos, com destaque para as resoluções 251/1997 e 301/2000 do Conselho Nacional de Saúde, lei 10 973/2004, RDC 10/2015 e RDC 9/2005 (13-17). A resolução 9/2015 (17) regulamenta a realização de ensaios clínicos com medicamentos para fins de registro. Também define os procedimentos e os requisitos para a realização desses ensaios clínicos que tenham a finalidade de fornecer subsídios para novas indicações terapêuticas; novas vias de administração; novas concentrações; novas formas farmacêuticas; ampliação de uso; nova posologia; novas associações; ou qualquer alteração pós-registro que necessite de dados clínicos, incluindo renovação de registro.

No Canadá, os ensaios clínicos são regidos pela Regulamentação de alimentos e medicamentos (*Food and Drug Regulations*), que teve sua última alteração em abril de 2019 (18). A resolução é composta por 10 partes (de A a J), sendo a parte C direcionada a medicamentos. Essa seção, por sua vez, também possui 10 divisões, sendo a quinta exclusiva para medicamentos em ensaios clínicos envolvendo seres humanos, com objetivo de regulamentar a venda ou a importação de medicamentos para ensaios clínicos envolvendo seres humanos, desde que a pesquisa esteja permitida no país.

Na Índia, o desenvolvimento de ensaios clínicos é regulamentado por uma norma de 1945 (*Drugs and cosmetic rules*) (19), que foi alterada em 1998 e também passou por consulta pública em 2005. Essa regulamentação possui 19 partes e diversos apêndices e anexos; o anexo Y aborda os requisitos e diretrizes para a realização de ensaios clínicos, e a parte XA aborda a importação de insumos para produção de novos medicamentos para ensaio clínico ou *marketing*.

A União Europeia possui a regulamentação 536/2014 do Parlamento Europeu (20), que aborda ensaios clínicos de medicamentos para uso humano. Nela, são apresentados aspectos relativos à investigação da eficácia e da segurança dos medicamentos para seres humanos.

Uma comparação entre os países quanto a aspectos da regulamentação aparece na tabela 1. Iniciando com as semelhanças, vale destacar que todos os países estudados exigem que os promotores (ou patrocinadores ou investigadores) se responsabilizem pelos estudos, cumprindo as BPC e tendo aprovação dos ensaios pelas autoridades sanitárias e conselhos de ética em pesquisa (17-20). Também são obrigados a elaborar relatórios em diversas fases dos estudos, fornecer informações adicionais, solicitar autorização para alterar substancialmente um estudo e para importar novos medicamentos (17-20). A União Europeia e o Brasil permitem que, caso o promotor não esteja estabelecido no país, um representante legal seja responsável pelo ensaio clínico (17, 20). As exigências básicas sobre rotulagem e taxas a serem pagas, o monitoramento e notificação dos efeitos adversos ocorridos e a necessidade de assegurar assistência médica aos participantes durante e após os ensaios clínicos também fazem parte das semelhanças os países estudados (17-20).

**TABELA 1.** Informações sobre regulamentação de ensaios clínicos em quatro países, 2019

Categoria de análise	Brasil	Canadá	Índia	União Europeia
Responsabilidades de patrocinador, promotor, investigador				
Notificação obrigatória	Inconformidades, auditorias e relatórios.	Inconformidades, auditorias e relatórios.	Inconformidades, auditorias e relatórios.	Inconformidades, auditorias e relatórios.
Assistência médica	Aos participantes, durante e após os ensaios clínicos.	-	Aos participantes, durante e após os ensaios clínicos.	Aos participantes, durante e após os ensaios clínicos.
Representante legal como responsável	Possível se o promotor não estiver estabelecido no país.	-	Possível se o promotor não estiver estabelecido no país.	Possível se o promotor não estiver estabelecido no país.
Documentação				
Autorização	Autoridade sanitária e Comitê de Ética em Pesquisa (CEP).	Autoridade sanitária e CEP.	Autoridade sanitária e CEP.	Autoridade sanitária e CEP.
Exigências básicas	Rotulagem e taxas a serem pagas.	Rotulagem e rastreabilidade dos medicamentos.	Rotulagem e taxas a serem pagas.	Rotulagem e rastreabilidade dos medicamentos.
Justificativa legal e comitê de ética	-	-	É necessária justificativa para realizar ensaio clínico no país. Alterações devem ser aprovadas pelo CEP.	Autorização direcionada ao(s) país(es) de interesse.
Qualidade e inspeção				
Boas práticas clínicas (BPC)	Deve cumprir BPC e assegurar proteção e assistência médica aos participantes.	Deve cumprir BPC e assegurar proteção e assistência médica aos participantes.	Deve cumprir BPC e assegurar proteção e assistência médica aos participantes.	Deve cumprir BPC e assegurar proteção e assistência médica aos participantes.
Diretrizes de qualidade	Documento das Américas.	-	-	Conferência Internacional em Harmonização (ICH).
Inspeção	Inspeções da autoridade sanitária podem ocorrer sem aviso.	Autoridade sanitária pode solicitar informações adicionais.	-	Inspeções da autoridade sanitária podem ocorrer sem aviso.
Prazos				
Início, suspensão, cancelamento e efeitos adversos	Na ausência de resposta da autoridade sanitária, desde que o CEP aprove é permitido iniciar o ensaio. Define prazos máximos (depois da identificação do problema) para suspender, cancelar e apresentar efeitos adversos.	Na ausência de resposta da autoridade sanitária, desde o CEP aprove é permitido iniciar o ensaio. Apresenta os prazos máximos (depois da identificação do problema) para suspender, cancelar e apresentar efeitos adversos.	Na ausência de resposta da autoridade sanitária, desde o CEP aprove é permitido iniciar o ensaio. Apresenta os prazos máximos (depois da identificação do problema) para suspender, cancelar e apresentar efeitos adversos.	Na ausência de resposta da autoridade sanitária, desde que o CEP aprove é permitido iniciar o ensaio. Apresenta os prazos máximos (depois da identificação do problema) para suspender, cancelar e apresentar efeitos adversos.
Entrega de relatórios	Define prazos máximos para entrega de relatórios (parciais e totais).	-	Define prazos máximos para entrega de relatórios (parciais e totais).	-
Aprovação de realização de ensaio clínico	Prazo máximo de 180 dias.	Prazo máximo de 30 dias.	-	Prazo máximo de 30 dias.
Armazenamento de arquivos	Tempo mínimo de 5 e 2 anos para ensaio clínico finalizado e descontinuado, respectivamente.	Tempo mínimo de 20 anos para ensaio clínico finalizado.	-	Tempo mínimo de 25 anos para ensaio clínico finalizado.
Alterações, modificações e emendas				
Implementação	Alterações não substanciais podem ser implementadas e apresentadas nos relatórios de acompanhamento. Alterações substanciais solicitadas: 1) pela autoridade sanitária devem ser implementadas imediatamente; 2) pelo promotor devem ser autorizadas pela autoridade sanitária antes de sua implementação.	Alterações não substanciais podem ser implementadas. A autoridade sanitária tem um prazo para negá-las. Se novos estudos comprovam benefícios terapêuticos para os seres humanos, a proibição da autoridade sanitária sobre o uso do medicamento pode ser revogada.	Preferencialmente devem ocorrer apenas para eliminar riscos de imediato. Caso ocorram, devem ser notificadas à autoridade sanitária e aprovadas pelo CEP.	Alterações substanciais solicitadas pelo promotor devem ser autorizadas pela autoridade sanitária antes de sua implementação. Estados-membros devem receber as informações, mesmo que não tenha participantes.
Suspensão e cancelamento				
Motivos e justificativas	Por parte do promotor, podem ocorrer em qualquer momento, desde que apresentadas as justificativas técnico-científicas e um plano de acompanhamento dos participantes. Por parte da autoridade sanitária, podem ocorrer quando as BPC não forem seguidas, ou havendo risco para a saúde e a segurança dos pacientes. Neste caso, os motivos devem apresentados.	Por parte do promotor, podem ocorrer em qualquer momento, desde que apresentadas as justificativas técnico-científicas e um plano de acompanhamento dos participantes. Por parte da autoridade sanitária, podem ocorrer quando as BPC não forem seguidas, ou havendo risco para a saúde e a segurança dos pacientes. Neste caso, os motivos devem apresentados.	Por parte do promotor, podem ocorrer em qualquer momento, desde que apresentadas as justificativas técnico-científicas e um plano de acompanhamento dos participantes. Por parte da autoridade sanitária, podem ocorrer quando as BPC não forem seguidas, ou havendo risco para a saúde e a segurança dos pacientes. Neste caso, os motivos devem apresentados.	Por parte do promotor, podem ocorrer em qualquer momento, desde que apresentadas as justificativas técnico-científicas e um plano de acompanhamento dos participantes. Por parte da autoridade sanitária, podem ocorrer quando as BPC não forem seguidas, ou havendo risco para a saúde e a segurança dos pacientes. Neste caso, os motivos devem apresentados.
Procedimentos após cancelamento do ensaio	Ensaios clínicos suspensos e/ou cancelados somente serão reativados após justificativas e aprovação da autoridade sanitária.	Ensaios suspensos também devem apresentar o impacto sobre os ensaios clínicos no país. Drogas não utilizadas devem ser recolhidas e descartadas.	Se a comercialização do medicamento é retardada após sua aprovação, deve-se informar a razão e quando o produto será comercializado.	-
Efeitos adversos				
Procedimentos caso ocorram efeitos adversos	Todas as reações adversas inesperadas devem ser monitoradas e relatados à autoridade sanitária. Tratamentos (de curto e longo prazo) devem ser implementados para proteger os participantes do ensaio clínico.	Todas as reações adversas inesperadas devem ser monitoradas e relatados à autoridade sanitária. Tratamentos (de curto e longo prazo) devem ser implementados para proteger os participantes do ensaio clínico.	Todas as reações adversas inesperadas devem ser monitoradas e relatados à autoridade sanitária. Tratamentos (de curto e longo prazo) devem ser implementados para proteger os participantes do ensaio clínico.	Todas as reações adversas inesperadas devem ser monitoradas e relatados à autoridade sanitária. Tratamentos (de curto e longo prazo) devem ser implementados para proteger os participantes do ensaio clínico.
Impacto no ensaio clínico	Servem para reavaliar os riscos e benefícios aos pacientes e rever a autorização da realização do ensaio clínico.	Servem para reavaliar os riscos e benefícios aos pacientes e rever a autorização da realização do ensaio clínico.	-	Os tratamentos devem ser direcionados para os ensaios clínicos de maior intervenção.
Instalações dos centros de ensaio clínico para fabricação				
Adequação	Estrutura física, equipamentos, instrumentos e recursos humanos devem estar adequados à condução do protocolo e atender as BPC.	Estrutura física, equipamentos, instrumentos e recursos humanos devem estar adequados à condução do protocolo e atender as BPC.	Estrutura física, equipamentos, instrumentos e recursos humanos devem estar adequados à condução do protocolo e atender as BPC.	Estrutura física, equipamentos, instrumentos e recursos humanos devem estar adequados à condução do protocolo e atender as BPC.
Especificações	-	-	-	Devem-se especificar os tipos e formas farmacêuticas do medicamento experimental fabricado e as operações de fabrico.
Importação				
Permissão	Necessidade de permissão para importar novos medicamentos. Se as condições de importação não forem seguidas, o estudo pode ser suspenso ou cancelado.	Necessidade de permissão para importar novos medicamentos. Se as condições de importação não forem seguidas, o estudo pode ser suspenso ou cancelado.	Necessidade de permissão para importar novos medicamentos. Se as condições de importação não forem seguidas, o estudo pode ser suspenso ou cancelado.	Necessidade de permissão para importar novos medicamentos. Se as condições de importação não forem seguidas, o estudo pode ser suspenso ou cancelado.
Relatórios				
Elaboração	Relatórios parciais e total devem ser elaborados, contendo as alterações ocorridas no período.	Relatórios parciais e total devem ser elaborados, contendo as alterações ocorridas no período.	Relatórios parciais e total devem ser elaborados, mesmo após o produto estar no mercado.	Relatórios parciais e total devem ser elaborados, e atualizados continuamente.
Supervisão	Comitês Independentes de Monitoramento de Segurança devem ser criados para acompanhar o desenvolvimento de ensaio clínico.	-	Novos estudos especificamente planejados ou conduzidos para examinar uma questão de segurança devem ser descritos.	Relatórios de países terceiros relativos ao ensaio clínico também devem ser apresentados aos Estados-membros.

Quanto às distinções entre a regulamentação brasileira e as dos demais casos, aponta-se a existência de várias resoluções brasileiras sobre o tema, enquanto os demais países concentram suas informações sobre ensaios clínicos em uma única regulamentação nacional. Na categoria documentação, as regulamentações brasileira e indiana não exigem rastreabilidade dos medicamentos (17, 18), com a regulamentação brasileira também não solicitando amostras (17). Entende-se por rastreabilidade a capacidade da autoridade sanitária (ANVISA, no caso brasileiro) de rastrear e localizar todos os medicamentos utilizados para ensaios clínicos.

No Canadá e na União Europeia, exigem-se recolhimento e descarte dos medicamentos não utilizados quando um estudo é suspenso ou cancelado (18, 20). Além disso, no Canadá e na Índia, os responsáveis devem apresentar o impacto (ou razões) do cancelamento (ou não comercialização) dos ensaios clínicos (ou medicamentos) (18, 19). Nenhuma dessas ações é aplicada no Brasil (17).

Na Índia, uma distinção positiva é a de que, para substâncias descobertas no próprio país, as fases I, II e III dos ensaios clínicos devem ser realizadas no próprio país (19). Para a União Europeia, os ensaios clínicos devem ser feitos preferencialmente com medicamentos auxiliares autorizados (20), mas, igualmente ao Canadá, o uso de medicamentos não autorizados pode ser permitido, desde que sejam comprovados benefícios terapêuticos para os seres humanos (18). A Índia e a União Europeia ainda permitem a não realização de estudos clínicos localmente caso já tenham sido feitos em outros países de acordo com normas éticas e com as BPC (19, 20), o que não ocorre no Brasil (17).

Na categoria prazos, a regulamentação brasileira difere das demais em dois itens críticos: tempo de aprovação mínimo para início do ensaio (180 dias no Brasil *versus* 30 dias na UE e no Canadá) e tempo de armazenamento dos arquivos relativos aos ensaios clínicos (até 5 anos no Brasil e no mínimo 20 anos nos outros países) (11, 17, 20).

Em relação aos efeitos adversos, no Brasil, todas as despesas relacionadas com procedimentos e exames (planejadas ou inesperadas) devem ser custeadas pelos responsáveis dos ensaios clínicos (11). Essas despesas, na UE, restringem-se aos ensaios clínicos mais invasivos (20).

Em relação ao cumprimento das BPC, enquanto os países da América Latina devem seguir o Documento das Américas (17), países europeus e o Canadá utilizam a Conferência Internacional em Harmonização (20). Por fim, apenas a regulamentação europeia aponta a necessidade de melhorias contínuas e atualização periódica da regulamentação (20).

## IMPLICAÇÕES PARA O CENÁRIO BRASILEIRO

Com base nas distinções detectadas entre os países analisados, é possível fazer algumas inferências, que apontam prejuízos no caso do Brasil. Inicialmente, o grande número de resoluções brasileiras pode dificultar a compreensão e o acesso à informação das indústrias farmacêuticas e demais interessados, afastando-os do país.

Da mesma forma, há prejuízos pela ausência de exigência quanto amostras e rastreabilidade dos produtos, recolhimento e descarte dos medicamentos não utilizados, ou explicitação dos motivos do cancelamento dos ensaios clínicos ou não comercialização dos medicamentos testados. A solicitação de amostra e a rastreabilidade de produtos permitiriam um controle de qualidade de ensaios clínicos mais eficaz (referente a efeitos adversos, cancelamento e suspensões dos estudos), bem como facilitaria o recolhimento dos produtos pela autoridade sanitária. Além disso, ao se eximir dessa responsabilidade, o governo brasileiro transfere-a aos promotores do ensaio clínico.

Outra observação diz respeito a ensaios suspensos ou cancelados e medicamentos não comercializados. Um ensaio clínico é cancelado caso apresente problemas, como falta de mercado, erros durante o ensaio ou efeitos adversos graves que a indústria desejaria esconder. Ao solicitar tais informações, a autoridade sanitária pode tomar as medidas cabíveis e garantir a segurança dos ensaios clínicos realizados no país. Além disso, o longo prazo para aprovação de ensaios clínicos no Brasil é um entrave, uma vez que amplia o risco operacional associado ao processo de P&D, estende o tempo para início do retorno sobre o investimento e desestimula a realização de testes clínicos no país. Como muitos estudos são conduzidos concomitantemente em vários países, atrasos no processo de aprovação podem limitar a participação brasileira nesses estudos (21).

Igualmente, entende-se como prejudicial o curto prazo para armazenamento de arquivos relativos aos ensaios clínicos. Por avaliarem princípios ativos que serão testados em seres humanos e que podem apresentar problemas em longo prazo (10 ou 20 anos), entende-se que o arquivamento da documentação, no Brasil, deveria ser ampliado, a fim de garantir a segurança da população.

Ainda, é distinta na legislação brasileira a responsabilidade dada ao patrocinador de custear todas as despesas relacionadas a ensaios clínicos, possivelmente com o objetivo de eximir o governo, e particularmente o Sistema Único de Saúde (SUS), dos custos médicos provenientes de ensaios clínicos. Ao mesmo tempo que é uma obrigatoriedade válida, visto os poucos recursos nacionais existentes e entendendo que as complicações foram causadas por um agente externo (promotor do ensaio clínico), também deve-se lembrar que nem todas as complicações geradas em ensaios clínicos são graves ou não atendidas pelo SUS.

Dessa forma, uma solução que contemplasse o uso adequado dos recursos brasileiros, garantindo que as indústrias farmacêuticas custeassem os gastos de efeitos adversos dos ensaios, poderia seguir o modelo da União Europeia: exigir apenas o custeamento de despesas relacionadas aos ensaios clínicos mais invasivos. Isso manteria a proteção dos sujeitos de pesquisa, mas reduziria os custos das indústrias farmacêuticas. Consequentemente, entende-se que seus interesses pelo país aumentariam, gerando desenvolvimento tecnológico e econômico, sem prejuízo à saúde da população ou ao erário público (21). Para esse fim, destaca-se a necessidade de atualização periódica da legislação brasileira, como ocorre na União Europeia, para permitir melhorias contínuas e contribuir para o progresso da ciência, da segurança e da saúde da população.

Finalmente, foram distinções consideradas positivas nos outros países: a necessidade de realização de ensaios clínicos para substâncias descobertas localmente (Índia); a permissão do uso de medicamentos auxiliares não autorizados, caso comprovado benefício para a população (União Europeia e Canadá); e a permissão para utilizar dados de estudos clínicos feitos em outros países (Índia e UE). Infere-se que a incorporação dessas exigências na legislação brasileira poderia estimular a busca por princípios ativos em território nacional, beneficiar suas pesquisas puras e seu desenvolvimento tecnológico. Além disso, ao permitir a continuidade de estudos que já ocorreram em outros países, o Brasil poderia adentrar mais facilmente nessa área da fronteira do conhecimento.

Entre as limitações deste estudo, destaca-se a comparação de apenas quatro casos, limitando a proposição de melhorias à política regulatória brasileira. Entre os pontos fortes do artigo, está a reunião de informações que antes estavam dispersas em diversos sites, explicando um tema de interesse internacional. Por isso, como recomendações, novos estudos poderiam comparar a regulamentação brasileira com os países que mais receberam ensaios clínicos nos últimos anos, utilizando as mesmas categorias de análise para ampliar a comparação.

## Contribuição dos autores.

WB concebeu a ideia original, interpretou os resultados e revisou o artigo. FSA e EDC coletaram e analisaram os dados, interpretaram os resultados e escreveram e revisaram o artigo. Todos os autores revisaram e aprovaram a versão final.

## Declaração.

As opiniões expressas no manuscrito são de responsabilidade exclusiva dos autores e não refletem necessariamente a opinião ou política da RPSP/PAJPH ou da Organização Pan-Americana da Saúde (OPAS).
